# The impact of local population genetic background on the spread of the selfish element *Medea‐1* in red flour beetles

**DOI:** 10.1002/ece3.5946

**Published:** 2019-12-19

**Authors:** Sarah A. Cash, Michael A. Robert, Marcé D. Lorenzen, Fred Gould

**Affiliations:** ^1^ Graduate Program in Genetics Department of Biological Sciences North Carolina State University Raleigh North Carolina; ^2^ W. M. Keck Center for Behavioral Biology North Carolina State University Raleigh North Carolina; ^3^ Department of Mathematics, Physics, and Statistics University of the Sciences Philadelphia Pennsylvania; ^4^ Department of Entomology and Plant Pathology North Carolina State University Raleigh North Carolina; ^5^ Genetic Engineering and Society Center North Carolina State University Raleigh North Carolina

**Keywords:** gene drive, maternal effect, *Medea*, selfish genetic element

## Abstract

Selfish genetic elements have been found in the genomes of many species, yet our understanding of their evolutionary dynamics is only partially understood. A number of distinct selfish *Medea* elements are naturally present in many populations of the red flour beetle (*Tribolium castaneum*). Although these *Medea* elements are predicted by models to increase in frequency within populations because any offspring of a *Medea*‐bearing mother that do not inherit at least one *Medea* allele will die, experiments demonstrating an increase in a naturally occurring *Medea* element are lacking. Our survey of the specific *Medea* element*,* M^1^, in the United States showed that it had a patchy geographic distribution. From the survey, it could not be determined if this distribution was caused by a slow process of M^1^ colonization of discrete populations or if some populations lacked M^1^ because they had genetic factors conferring resistance to the *Medea* mechanism. We show that populations with naturally low to intermediate M^1^ frequencies likely represent transient states during the process of *Medea* spread. Furthermore, we find no evidence that genetic factors are excluding M^1^ from US populations where the element is not presently found. We also show how a known suppressor of *Medea* can impair the increase of M^1^ in populations and discuss the implications of our findings for pest‐management applications of *Medea* elements.

## Graphical abstract

“The impact of local population genetic background on the spread of the selfish element Medea‐1 in red flour beetles” is a laboratory study that aims at determining whether field populations that lack Medea or have intermediate frequencies of Medea have some degree of genetic resistance to the selfish mechanism that causes Medea to spread.

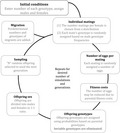

## 1 INTRODUCTION

Since the first selfish genetic elements (SGEs) were discovered nearly a century ago (Gershenson, 1928), they have been described in a wide variety of organisms, yet their evolutionary implications are only partially understood and practical uses are just now being explored (Burt & Trivers, 2006; Macias, Ohm, & Rasgon, 2017; Piaggio et al., 2017). SGEs are inherited more frequently than expected by Mendelian inheritance as a result of mechanisms that either kill the alternative allele, increase the element's own replication, or preferentially segregate the element into gametes during meiosis (Burt & Trivers, 2006). They are able to increase in frequency by eschewing the laws of inheritance, and they can affect the evolutionary trajectory of a population by impacting the fitness of individuals carrying the elements (typically negatively, though some examples of the positive fitness effects of SGEs exist) and by spreading linked, hitchhiking alleles (Fishman & Kelly, 2015; Werren, Nur, & Wu, 1988).

In one such element, *Medea*, any offspring of a *Medea*‐bearing mother that do not inherit at least one *Medea* allele will die (Beeman, Friesen, & Denell, 1992). This results in an evolutionary advantage of individuals with Medea elements over those without this genetic element. Population genetic models predict that *Medea* will rapidly increase in frequency in a population under a broad array of ecological parameters (Akbari et al., 2014; Chen et al., 2007; Hastings, 1994; Smith, 1998; Wade & Beeman, 1994; Ward et al., 2010). Beyond interest in *Medea* elements to further our understanding of evolution, they are of interest for potential use in genetic pest management. Through linking an antipathogen construct to a *Medea* element, the combined construct could presumably be driven to fixation in a pest population, rendering that population incapable of vectoring disease (reviewed in Hay et al., 2010; Sinkins & Gould, 2006). The deterministic, theoretical models of *Medea* have been able to predict how synthetic *Medea* elements spread within homogeneous laboratory populations of *Drosophila* species (Buchman, Marshall, Ostrovski, Yang, & Akbari, 2018; Chen et al., 2007) but it is not clear that such models will be capable of predicting behavior of naturally occurring *Medea* in genetically diverse natural populations.

While *Medea* elements utilized in genetic pest management will likely be synthetic, a better understanding of the dynamics of naturally occurring *Medea* elements in genetically diverse populations could enable more accurate predictions of the behavior of engineered *Medea* elements if, in the future, they are released in the environment. Red flour beetle (*Tribolium castaneum*) populations (Beeman & Friesen, 1999) contain a number of natural *Medea* elements. Two of these, *Medea‐1* (M^1^) and *Medea‐4* (M^4^), have been detected in many populations of red flour beetle across the globe. Although both possess maternal‐effect lethality, the elements map to opposite ends of chromosome 3 and do not cross rescue (i.e., inheritance of an M^1^ allele does not rescue the lethality imposed by an M^4^‐bearing mother, and vice versa; Beeman & Friesen, 1999). Although the exact mechanism of this lethality is unknown, the current model of a Medea element includes two tightly linked loci which encode (a) a maternally expressed toxin deposited in all eggs and (b) a zygotic antidote that rescues only offspring inheriting at least one Medea allele (Beeman & Friesen, 1999; Beeman et al., 1992). While M^1^ and M^4^ are distinct genomic elements, they share an incompatibility with another genomic region, the hybrid incompatibility factor (*H*) (Thomson & Beeman, 1999). Located on chromosome 9, H is fully incompatible with M^1^, resulting in the death of all offspring produced from a pairing between M^1^ and H. The interaction between H and M^4^ is less severe, and viable offspring may be produced in some crosses at specific temperatures (Thomson, 2014; Thomson & Beeman, 1999).

A survey of M^4^ in the United States from 1993 through 1995 suggested existence of a latitudinal boundary for its spread. Most populations above 33°N were fixed for the element, while most populations sampled below this latitude lacked the M^4^ element altogether (Beeman, 2003). Our recent survey work demonstrates that M^4^ has spread beyond this boundary, but many populations still lack M^4^ (Cash, Lorenzen, & Gould, 2019). Similarly, for the *Medea* element M^1^, an assessment of beetles collected in 2004–2007 and those collected by us from 2012 through 2014 indicates a patchy distribution within the United States (Cash et al., 2019). From the surveys, we could not determine the causes of the patchy distribution, which could include ongoing, slow stochastic spread of the elements among populations or population‐level resistance to the *Medea* drive mechanism.

In the present study, we conducted a series of laboratory experiments to better understand the cause(s) of the current distribution of M^1^ in United States populations and to more generally understand the dynamics of natural *Medea* elements. We focused on M^1^ instead of M^4^ because we had a genetic marker within the M^1^ sequence, and this made monitoring of frequencies efficient. Although dynamics of synthetic *Medea* constructs in laboratory populations of *Drosophila* species have been monitored (Akbari et al., 2014; Buchman et al., 2018; Chen et al., 2007), multigenerational studies of natural *Medea* elements have not yet been reported. Unlike previous laboratory studies that were restricted to genetically homogenous test populations, we used wild‐derived flour beetle populations to assess how *Medea* would spread in genetically diverse populations and whether some populations are resistant to *Medea's* mechanism for spread. We tested a number of hypotheses by comparing our results against predictions of a stochastic model of red flour beetle population dynamics and genetics that we specifically designed to match the demography and genetics of our experimental populations (Appendix S2).

We test the following hypotheses:

Hypothesis 1 In wild populations with intermediate frequencies of M^1^, the intermediate frequency is transitional, and therefore the frequency of M^1^ will increase over generations when samples of these populations are maintained under laboratory conditions. (Alternate hypothesis: M^1^ frequency will not increase in the laboratory because M^1^ could not function within the genome of these wild populations and was actually maintained at intermediate frequency as a neutral allele in the wild populations.)

Hypothesis 2 Wild populations that lack M^1^ have an incompatibility factor that prevents M^1^ from establishing. Therefore, M^1^ artificially introduced into these populations in the laboratory will not increase in frequency or will increase more slowly than they do in populations that were initially fixed for M^1^ in the wild. (Alternative hypothesis: Lack of M^1^ in populations was simply due to chance, and rate of increase would be the same in both populations that did and did not have M^1^ in the wild.)

Hypothesis 3 The known hybrid incompatibility factor, H, will inhibit increase in frequency of M^1^, and high frequency of M^1^ will cause a decline in H frequency.

## 2 MATERIALS AND METHODS

### 2.1 Population maintenance

For all studies, beetles were kept under constant dark conditions at 30°C and 58% (±2%) relative humidity on a mixture of 1:20 by volume Brewer's yeast to organic whole wheat pastry flour. Both laboratory strains and wild‐derived strains were used in this study (Table 1).

**Table 1:** Origins and genotypes of *Tribolium castaneum* strains

Strain name	Origin/description	Genotype
Laboratory strains		
GA‐1	Georgia, USA (1980; Haliscak & Beeman, 1983)	Wild‐type
*ab*	Colombia (1980; Vasquez & Del Castillo, 1985)	M^1^, M^4^
IPS	*ab* and GA‐1 cross, followed by 8 generations of random mating	M^1^, M^4^
*pearl*	Park (1937)	M^4^
Pig‐19	An M^1^, M^4^ bearing stock created in a *pearl* background (Lorenzen et al., 2003)	M^1^, M^4^
M^1^	Pig‐19 and GA‐1, selection for non‐M^4^ progeny (Figure 4)	M^1^
Bha‐G (iso6)	Indian origin; acquired from M.S. Thomson (2011)	H
Wild‐derived strains		
LA‐4	Acadia Parish, Louisiana (2012)	M^1^ a, M^4^
TN‐3	Obion County, Tennessee (2012)	M^1^ a, M^4^
TX‐3	Hale County, Texas (2012)	M^1^ a, M^4^
AL‐11	Fayette County, Alabama (2012)	M^1^, M^4^
OH‐1	Seneca County, Ohio (2011)	M^1^, M^4^
MS‐1	Marshall County, Mississippi (2012)	M^1^, M^4^
AL‐9	Henry County, Alabama (2012)	Wild‐type
ND‐1	Grand Forks County, North Dakota (2011)	M^4^

a *Medea* element is present at an intermediate frequency.

### 2.2 M^1^ genotyping

Genomic DNA from individual beetles was extracted using the method described by Lagisz, Port, and Wolff (2010). One µl of the resulting DNA solution was used in a 25 µl PCR consisting of 1X Buffer, 4 mM MgCl_2_, 0.2 mM dNTPs, 1 mM forward primer, 0.5 mM of each reverse primer, and 1 U *Taq* polymerase (Genesee). Primers used for amplification were as follows:

Forward primer: 5′‐TGGCGATAGTCAAAATCCTTTGTCG‐3′

M^1^ Reverse: 5′‐TGCCACCTTCACGTAGCCCG‐3′

Wild‐type Reverse: 5′‐CAGGGCCCCGGAGTATTTTTCC‐3′

Alleles were separated on 2.5% agarose gels infused with ethidium bromide and visualized by ultraviolet (UV) illumination.

### 2.3 Dynamics of M^1^ in colonies from populations that had intermediate frequencies

Experimental populations were developed from field‐caught adults collected from natural populations that had intermediate frequencies of M^1^. Samples used in this study (LA‐4, TN‐3, and TX‐3) were collected in 2012 and genotyped for the M^1^ element (Cash et al., 2019).

Two replicates of each population were established and reared under a discrete generation regime, each with 200 randomly selected field‐caught adults. These beetles were placed on a fresh flour mixture in glass pint canning jars and permitted to mate and oviposit for one week before removal and genotyping of these original adults. After five weeks, 200 offspring were selected at random to parent the next generation, and of these, roughly 50 individuals per replicate per generation (average = 50 ± 5 *SD*) were randomly selected for M^1^ genotyping after the one‐week mating period had ended. This process was repeated for eight generations. M^1^ genotyping was performed using the PCR primers and protocol described above.

### 2.4 Comparative M^1^ dynamics in populations that had been fixed for M^1^ or lacked M^1^

To assess the potential role of genetic background on M^1^ frequency dynamics, we investigated populations known to harbor M^1^ at high frequency as well as populations which lacked the element (and thus may have been able to resist or impair M^1^ spread). Of the populations of field‐caught beetles that we genotyped either as fixed for the M^1^ element or fixed for the wild‐type (non‐M^1^) allele, five were selected for this study: three M^1^‐fixed (“susceptible”) and two non‐M^1^ (potentially “resistant”) populations. Our laboratory strains were each derived from at least 50 field‐caught adults. Strains for the experiments were selected based on their location within the larger M^1^ distribution and on their health in laboratory culture. For example, two Alabama‐derived populations—one fixed for M^1^ and the other lacking the element—were selected because of their proximity to each other. An M^1^‐fixed Mississippi‐derived population was selected because it was situated within a large geographic swath of other M^1^‐fixed populations.

Field‐caught red flour beetles used in this study were collected between November 2011 and December 2012 and previously genotyped for M^1^ using a PCR marker as described above. Laboratory strains of known *Medea* status were employed in crosses to the field‐collected beetles. These were the non‐*Medea* GA‐1 strain (used as a source of wild‐type alleles) and IPS, a strain harboring both M^1^ and M^4^ elements, created by several generations of intermating the progeny of a cross between GA‐1 and the *ab* strain that is fixed for both M^1^ and M^4^.

#### 2.4.1 Assessing frequency changes in M^1^ “susceptible” populations

Virgin females from M^1^‐fixed populations (AL‐11, OH‐1, MS‐1) were crossed to GA‐1 males to generate M^1^ heterozygotes. Because all offspring of a mother with *Medea* will themselves inherit *Medea*, it was not possible for this study to generate a non‐M^1^ population derived from the source population that retained any significant portion of the original wild population genetic background. Thus, we set up study populations seeded entirely by M^1^ heterozygotes, created from crosses between homozygous M^1^ wild‐derived females and males of the GA‐1 strain that lack both M^1^ and M^4^. These M^1^ heterozygotes each carried approximately 50% of the M^1^‐fixed source population genetic background and 50% of the GA‐1 background. The GA‐1 genomic background was expected to be permissive for increase in M^1^ frequency based on previous crosses (Beeman et al., 1992). One hundred virgin heterozygotes (50 each males and females) derived from each of the three original M^1^‐fixed populations were selected at random, with four replicates of each population. These adults were allowed to mate and oviposit for one week in half‐pint glass jars with the flour–yeast mixture. The adults were then removed, sacrificed, and genotyped for the M^1^ element (see Figure S1 for details). After five weeks, 100 adult offspring were chosen at random to produce the next cohort; these adults were placed on fresh flour and removed after one week for genotyping. This process was repeated for five generations. M^1^ allele frequency was assessed each generation by genotyping approximately 50 individuals per replicate (average = 52.5 ± 7.5 *SD*) using the primers and PCR protocol described above.

#### 2.4.2 Assessing frequency changes in M^1^ “resistant” populations

The introduction of M^1^ into a “resistant” population genome first required the creation of a population of M^1^ individuals who carried as much of the “resistant” genetic background as possible from the AL‐9 or the ND‐1 strain.

Virgin females from a wild‐derived, non‐M^1^ source population were crossed to males from the IPS strain to generate heterozygous males. These males were backcrossed to females from the source population for three generations. After the third backcross, offspring were mated in single pairs, then sacrificed, and genotyped for M^1^ as described above. M^1^‐positive offspring were used as the source of M^1^ in introductions to non‐M^1^ “resistant” populations; for each source population, M^1^ was introduced at two allele frequencies, 0.25 and 0.5, with 100 adults per replicate (at equal sex ratios), and three replicates of each frequency. Population maintenance was the same as described for the “susceptible” populations.

### 2.5 Hybrid incompatibility factor crosses

#### 2.5.1 Creation of an M^1^‐only strain

The hybrid incompatibility factor (H) interacts with both M^1^ and M^4^. We chose to examine the multigeneration dynamics of H with M^1^ because this interaction is strong and bidirectional (incompatible regardless of which parent has the M^1^ genotype), so assessing the population‐level impact of H on M^1^ allele frequency is expected to be more straightforward than the same experiment using M^4^, where H interacts more weakly and in a unidirectional manner (incompatibility is less severe when the M^4^‐bearing parent is male; Thomson & Beeman, 1999).

Because we did not have an M^1^ strain that lacked M^4^, it was necessary to create our own. We selected the Pig‐19 strain (Lorenzen et al., 2003) as the source of M^1^ because it has performed well in crosses in our laboratory, and our cultures were in good health. GA‐1 females were crossed to Pig‐19 males to generate M^1^, M^4^ double‐heterozygotes, and males derived from this cross were backcrossed to GA‐1 females. Because M^1^ and M^4^ are located at opposite ends of the same chromosome, they are expected to recombine freely, such that this backcross produces four genotypes—M^1^, M^4^ double‐heterozygotes, M^1^‐heterozygotes, M^4^‐heterozygotes, and wild‐type offspring—in nearly equal proportions. Male offspring were crossed to M^4^‐heterozygote females to diagnose whether the male carried M^4^. Non‐M^4^ males were then mated to a GA‐1 female before being sacrificed and genotyped for M^1^. Only those crosses involving a male who lacked M^4^ but carried M^1^ were retained to create the M^1^‐only strain (see Figure S2 for details).

#### 2.5.2 Assessing the dynamics of M^1^ frequency in populations with H

Using individuals from our M^1^‐only strain, we created three populations, each seeded by 50 M^1^‐heterozygotes, along with 50 H‐homozygous individuals (Thomson & Beeman, 1999), all with 1:1 sex ratios. In these populations, the initial frequency of the H incompatibility factor was 0.5, while initial M^1^ allele frequency was 0.25. Three additional populations were created, again with 100 individuals per population but in this case with M^1^ and H homozygotes in equal numbers and 1:1 sex ratios. Population maintenance was the same as described for the “resistant” and “susceptible” populations, with M^1^ genotyping occurring in each of the three generations.

### 2.6 Estimating effective population size in experimental populations

In order to confirm that changes in our observed *Medea* frequencies were due to the inherent drive of the M^1^ allele, and not simply a result of genetic drift in these experimental populations, we estimated effective population size in the selected experimental populations. Both replicates of the intermediate M^1^ population LA‐4 were used, as well as one replicate each from intermediate populations TN‐3 and TX‐3, and one replicate from “susceptible” population MS‐1. From each of these, 40 individuals per population were selected at random from generation 0 and generation 6 (MS‐1 was tested at generation 1 and generation 5) and genotyped at four polymorphic microsatellite loci. Microsatellite genotyping methods were as described in Cash et al. (2019). Differences in observed temporal allele frequencies were used to estimate effective population size in the program MLNe (Wang, 2001; Wang & Whitlock, 2003). MLNe uses both a pseudo‐maximum‐likelihood approach as well as a moment estimator to estimate effective populations size based on allele frequency data collected at different generational time points. Because our experimental replicates are closed populations, migration was not considered in the estimation. We used a maximum N_e_ value of 10,000 for the estimates.

### 2.7 Modeling *Medea* in populations

**Figure 1:**
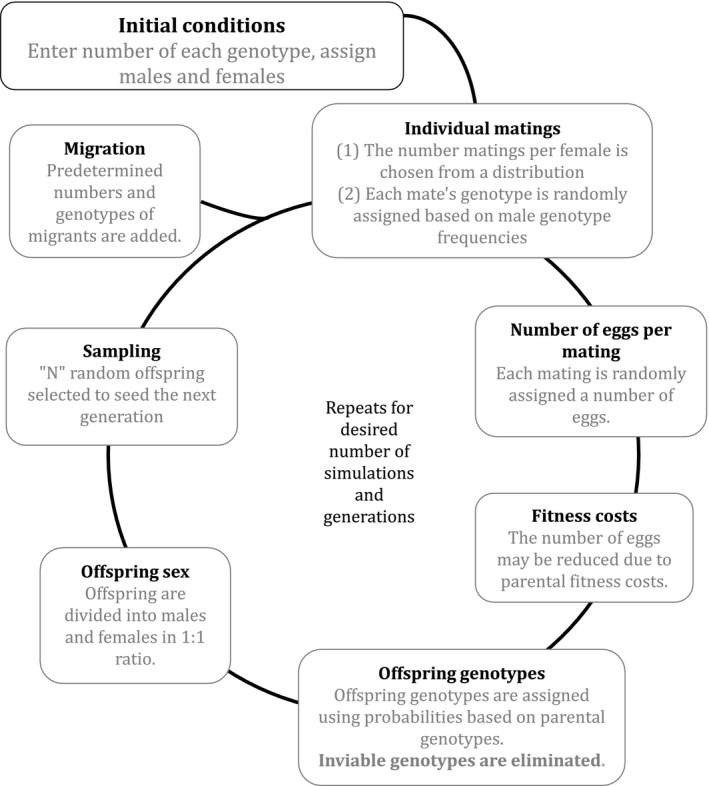
Our model of *Medea* population dynamics

We coded a stochastic model of *Medea* dynamics and used to make predictions about *Medea* frequency changes under each of the population regimes described above (Figure 1, for details see Appendix S2). Our individual‐based model first partitions *N* (population size) individuals into males and females and then into genotype categories, using predefined initial sex and genotype frequencies. Each female is randomly assigned a number of mates, based on a Poisson distribution of mate numbers. The number of eggs resulting from each mating is determined by selecting randomly from the empirical egg‐laying data described below. At this stage, the number of eggs may be decreased by a predetermined percentage, due to genotype‐specific fitness costs incurred by the parents based on presence of the *Medea* element itself, not to genomic interactions. Offspring are assigned genotypes based on the probabilities expected from each parental genotype combination. All offspring produced in a given generation are pooled by genotype, after which N are selected as parents of the next generation. At least 100 simulations of the model were run for each set of initial genotype frequencies.

In order to parameterize the model to make more accurate predictions about these particular populations, we generated additional data on reproductive fitness of female genotypes used in the experiments. Single‐pair crosses were set up between individuals of the key genotypes. After three days at 30°C, adults were removed, and eggs were counted. In total, eggs were censused from 328 unique crosses from a variety of parental genotypes, providing an overall egg‐production distribution, which was incorporated into the model. Most previous models of *Medea* dynamics assume that heterozygous offspring of homozygous *Medea* mothers have no fitness cost due what could be a lower dosage of *Medea*. However, results from a model by Ward et al. (2010) demonstrate that such a cost could have an impact on dynamics. We therefore performed additional tests to examine the fitness of the heterozygous offspring of homozygous *Medea* mothers. These experiments revealed that the heterozygous female offspring of homozygous M^1^ mothers had a nonsignificant trend of lower egg production and offspring survival, resulting in an approximated 30% lower production of surviving offspring (Cash, 2016 Appendix C). We incorporated this possibility into our model.

Although the M^4^ element was present in many of our experimental populations (Table 1), we do not currently have a reliable marker for M^4^; thus, we focused our genotyping efforts only on M^1^. Because models suggest that the presence of M^4^ may impact the rate of spread of M^1^ (Cash, 2016 Chapter 4), we incorporated estimates of initial M^4^ frequency based on diagnostic crosses (Cash et al., 2019) into our model for those populations likely to be harboring the M^4^ element.

## 3 RESULTS

### 3.1 The M^1^ frequency generally increased in colonies from populations that had intermediate frequencies (Hypothesis 1 supported)

**Figure 2:**
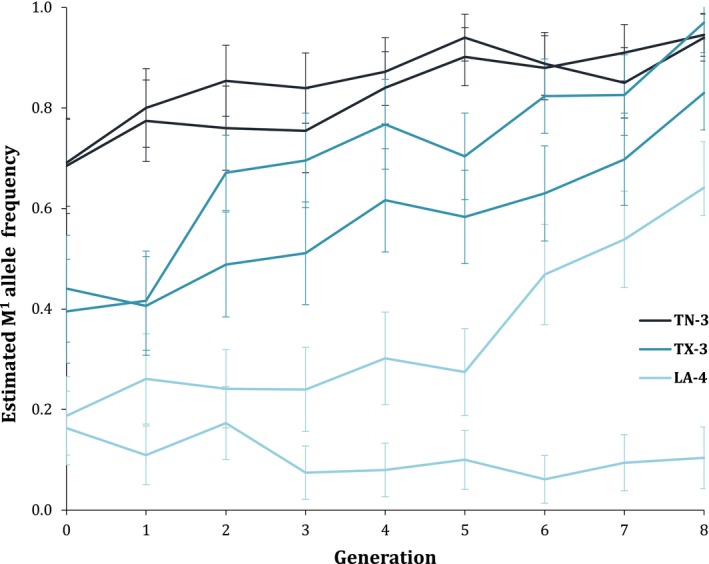
Estimated M^1^ allele frequency increased in most wild‐derived populations. 95% confidence intervals for each allele frequency measurement are shown

The M^1^ element generally increased in frequency in our laboratory colonies that were started from wild populations with intermediate M^1^ frequencies (Figure 2). In our lowest initial‐frequency population, LA‐4, one replicate increased in M^1^ frequency (from an estimated 0.188 to 0.642) over the course of eight generations, while the other decreased (from an estimated 0.163 to 0.104, with a low of 0.061). In the replicate colonies from our higher initial‐frequency populations, TN‐3 (initial M^1^ = 0.685, 0.691) and TX‐3 (initial M^1^ = 0.395, 0.440), both replicates of each population increased in M^1^ frequency over eight generations (Figure 2).

**Figure 3:**
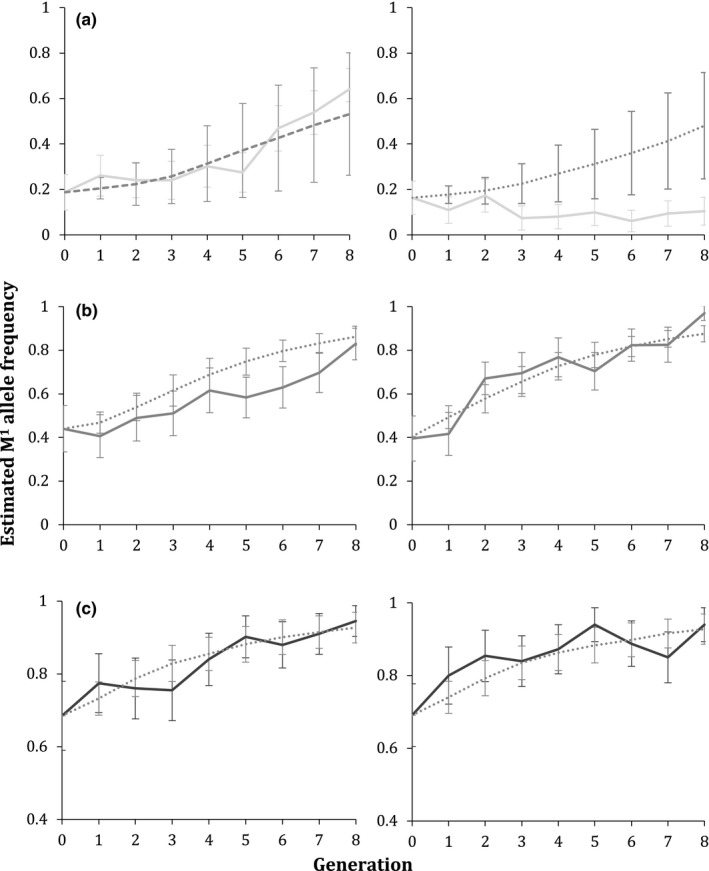
Experimental M^1^ element increases resemble model results. Solid lines show two replicates from the (a) LA, (b) TX, and (c) TN populations, and dotted/dashed lines represent the model that is the best visual fit to the data. For each, Replicate 1 is on the left, and Replicate 2 is on the right. Dotted lines represent models with heterozygote fitness cost of 0.3, while dashed lines represent models without these costs

Our model predicted the rate of M^1^ increase that occurred in 5 of our 6 experimental replicates. For higher initial starting frequencies of M^1^, fit of the model to the data slightly improved when fitness cost of heterozygous offspring of homozygous mothers of 0.3 was added to the model (see Figures 3 and S3).

### 3.2 Wild US populations that lacked M^1^ are not resistant to M^1^ increasing in frequency (Hypothesis 2 not supported)

**Figure 4:**
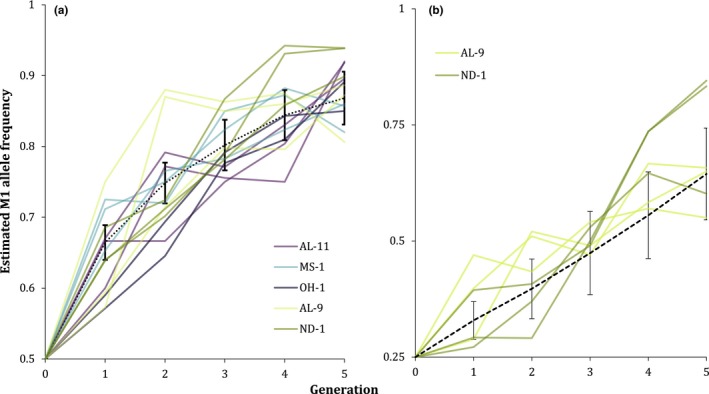
M^1^ frequency increases in both previously fixed and previously non‐M^1^ populations. (a) Previously fixed “susceptible” (yellow/green) and previously non‐M^1^ “resistant” (blue/purple) 0.5 initial‐frequency replicates compared to model expectations with a heterozygote fitness cost of 0.3. (b) Previously non‐M^1^ 0.25 initial‐frequency replicates compared to model expectations with no fitness cost

Not surprisingly, M^1^ increased rapidly within populations previously fixed for the element (Figure 4a). M^1^ also increased in each replicate of the previously M^1^‐free populations (Figure 4a,b). While the initial M^1^ frequencies were identical, the model predictions for the previously non‐M^1^ populations differ at the lower introduction frequency due to their expected M^4^ frequencies—ND‐1 appears to have a high M^4^ frequency, while AL‐9 seems to lack the element (Cash et al., 2019). When M^1^ is introduced into an M^4^‐fixed population (even at low frequencies), expectations of increase are similar to predictions of a single *Medea* introduced into a non‐*Medea* population (Cash, 2016). When two unlinked *Medea* elements (such as M^1^ and M^4^) are both at low frequency in a population, it is more likely that an element will be lost than in a single‐*Medea* introduction at that same frequency, as two independent elements incapable of cross‐rescue are present in small numbers.

Thus, the predictions for ND‐1 include an expectation of near‐fixation for M^4^ and a low level of M^4^ for AL‐9 (estimated at 0.125) due entirely to the introduction of the element during crossing to insert M^1^ into the AL‐9 genetic background. Although some caution should be used in directly comparing the results of the previously fixed populations (established as F_1_s from a cross between wild‐derived and laboratory stock) and the previously non‐M^1^ populations (established from backcrossing to maintain wild population genetic background), it is clear from visually comparing the two that the previously non‐M^1^ populations do not appear to be resistant to M^1^ increase.

As with results from the experiments using colonies from populations with intermediate frequencies, models including fitness costs for heterozygous offspring of homozygous mothers appear to be a slightly better visual fit at the higher initial frequencies (Figure S4a), whereas models without heterozygote fitness costs fit the data better at lower frequencies (Figure S4b).

### 3.3 The presence of the hybrid incompatibility factor does not always impair increase in M^1^ frequency (Hypothesis 3 not supported)

**Figure 5:**
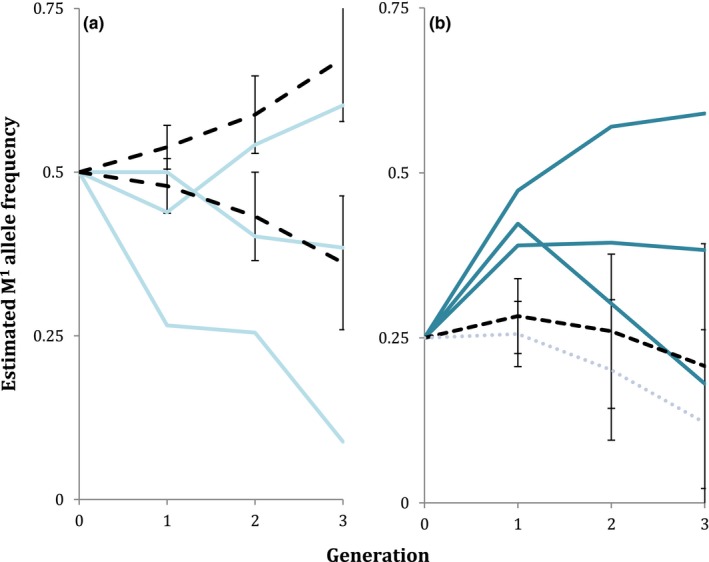
The presence of H results in increased variation in M^1^ population dynamics. (a) Solid lines show M^1^ frequency decreased in two of three replicates when both M^1^ and H were at initial frequencies of 0.5. Two model predictions (dashed lines) represent expectations when M^1^ either increases to fixation, eliminating H from the simulated population, or is lost in the presence of H. (b) The fate of M^1^ varied when M^1^ was introduced into an H‐bearing population at initial allele frequencies of 0.25 M^1^ and 0.5 H. Dashed lines are predictions from an M^1^‐H model with heterozygote‐associated fitness costs

As predicted, the hybrid incompatibility factor (H) hindered the increase in the M^1^ element when both were at a frequency of 0.5 (Figure 5). While one replicate did increase slightly overall, the negative impact of H is clear when comparing this replicate to the much more rapid increase seen in the non‐H wild US populations that lacked M^1^ and were set up at the same initial M^1^ frequency as populations with H. Several individuals in these replicates were heterozygous for M^1^. Because successful hybridization of M^1^ and H has not yet been demonstrated, this is likely the result of a lack of total homozygosity in the parental strains used.

At an introduction frequency of 0.25 M^1^, 0.5 H, however, the results were very different. In contrast to model predictions of M^1^'s decline, M^1^ frequency had actually increased in two of the three replicates after three generations. While the mean M^1^ frequency decreased, as expected by our model (with fitness costs), a frequency increase was predicted in over a quarter of simulations (Generation 3 M^1^ allele frequency >0.25 in 79/300 simulations). The initial increase in M^1^ frequency in each replicate is the result of an initially heterozygous M^1^ population. Because the parental generation lacks M^1^ homozygotes, *all* matings between M^1^‐bearing individuals will experience *Medea*‐dependent killing of wild‐type offspring (and their wild‐type alleles), and the M^1^ allele frequency will undergo an early bump upwards.

### 3.4 Estimates of effective population size in our experimental replicates were low

In some cases, estimates of effective population sizes were lower than the census population size (100 for MS‐1, 200 for other populations; Table 2), but the confidence limits for the estimates were typically large and in some cases the upper limit of the confidence interval exceeded the assumed highest population size. In order to ensure our model was capturing the extent of genetic drift occurring in our experimental populations, we also ran models using the lower, moment‐estimated effective population sizes—for example, the comparative model run for Replicate 1 of population LA‐4 simulated populations with the same initial starting M^1^ frequency, but with 31 individuals (instead of 200) randomly chosen to parent each successive generation (Figure S3a).

**Table 2:** Estimates of effective population size vary among experimental populations

Source population	Locus 4.7		Locus 5.13		Locus 6.18		Locus 9.24		ML Est. $\hat{N_{e}}$	Mom. Est. $\hat{N_{e}}$
	G_0_	G_6_	G_0_	G_6_	G_0_	G_6_	G_0_	G_6_		
LA‐4 (Rep1)	6	3 (2)	4	4 (4)	4	3 (3)	4	5 (4)	37 (19–88)	31
LA‐4 (Rep2)	6	6 (4)	4	4 (4)	4	4 (4)	4	5 (4)	164 (49–10,000)	85
TN‐3 (Rep2)	10	9 (9)	2	4 (2)	4	4 (4)	4	4 (4)	93 (36–689)	70
TX‐3 (Rep1)	6	6 (5)	3	3 (3)	4	4 (4)	4	4 (4)	184 (38–10,000)	130

Source population	Locus 4.7		Locus 5.13		Locus 6.18		Locus 9.24		ML Est. $\hat{N_{e}}$	Mom. Est. $\hat{N_{e}}$
	G_1_	G_5_	G_1_	G_5_	G_1_	G_5_	G_1_	G_5_		
MS‐1	7	8 (7)	4	3 (3)	4	4 (4)	4	2 (2)	193 (31–10,000)	134

Note

For each of the four polymorphic microsatellite loci examined, the number of alleles present in a sample of the parental generation G_0_ is shown, along with the number of alleles present in the sixth generation, G_6_ (the MS‐1 sample was taken during G_5_, as this was the final generation of the MS‐1 experiments). In parenthesis is the number of alleles shared between the two sampled time points. Both maximum‐likelihood (ML Est.; with 95% confidence intervals) and moment (Mom. Est.) estimates of effective population size $\left(\hat{\text{Ne}}\right)$ using the pseudotemporal method in MLNE (Wang, 2001; Wang & Whitlock, 2003) are shown. In several cases, the ML upper confidence limit reached 10,000, the maximum value set for this parameter, indicating that the program was unable to resolve the upper bound with our data.

## 4 DISCUSSION

Models of *Medea* and experiments with synthetic *Medea* constructs in *Drosophila* species predict that as long as there are no fitness costs, the element will increase in frequency rapidly once introduced into a non‐*Medea* population (Akbari et al., 2014; Buchman et al., 2018; Chen et al., 2007; Huang, Lloyd, Legros, & Gould, 2009; Smith, 1998; Wade & Beeman, 1994; Ward et al., 2010). For genetic pest management, these models are useful in predicting the effectiveness of a particular approach before costly constructs and strains are built and field trials are performed. But for these models to reflect biologically realistic scenarios, it is critical to have experimental data for model parameterization. Until now, no studies had examined the spread of natural *Medea* elements in insects from diverse wild populations. Here, we have examined three hypotheses that are critical for understanding potential for variation in parameters that influence *Medea* frequency changes in populations:

Hypothesis 1 Intermediate M1 frequency is transitional

We have presented the first evidence that the M^1^ element does indeed increase in frequency in red flour beetle populations and that the “selfish” behavior of the M^1^ element is functional in populations which had intermediate frequencies of M^1^ at the time of sampling. In one of the six replicates of intermediate frequency populations, the element decreased in frequency, but this is not unexpected when, as in this case, the element is at low frequency and the effective population size is small (Cash, 2016 Chapter 4; Ward et al., 2010). Caveat: Our experiments were conducted under laboratory conditions with discrete generations. There is a possibility that under natural environmental conditions, results would have differed.

Hypothesis 2 Wild populations that lack M1 have an incompatibility factor that prevents M1 from establishing

We have shown that M^1^ is capable of establishing in populations where it was previously absent, indicating that genetic background is likely not a major factor in excluding M^1^ from the populations examined here (Figure 4). In light of M^1^'s ease of spread within populations where it was previously absent, what explains the lack of M^1^ in the ND‐1 and AL‐9 populations? In a previous study, we found no evidence of population structure to suggest that these populations are completely cutoff from the rest of the M^1^‐bearing populations of the United States (Cash et al., 2019). Perhaps due to the nature of the facilities where the beetles were found, most migration is from populations where M^1^ is at low frequency, and thus presenting little opportunity for M^1^ to be introduced or to increase after a low‐frequency introduction. Low‐frequency clusters within the United States were identified in our recent survey (Cash et al., 2019), and in an earlier survey, it was clear that M^1^ was absent in some geographic areas. It is feasible that the absence in some populations could be due to the fact that beetles with M^1^ had by chance not colonized the sites. Follow‐up surveying in future years will be useful for examining this hypothesis.

Alternatively, while our study results do not suggest the presence of suppressors in these populations, it does not mean that they do not exist. It is possible that suppressors exist at low or intermediate frequency in these populations and were not captured by our random selection of parents to seed our backcrosses and initial generations. Caveat: If suppressors are present, but not fixed, they may prevent M^1^ from increasing at low frequency—perhaps our introduction frequencies (0.25 and 0.5) were too high to overcome. The potential impact of genetic background on *Medea* dynamics merits further investigation, including studies into whether the spread of M^4^ is impacted by other genetic factors. We currently lack a reliable genotyping marker for the M^4^ element; thus, we focus on M^1^ presently.

Hypothesis 3 The known hybrid incompatibility factor, H, will inhibit increase in frequency of M1, and high frequency of M1 will cause a decline in H frequency

While not a major factor for the previously naïve populations described above, we have shown that genetic background *can* influence M^1^ frequency, as the presence of the H factor results in variable M^1^ dynamics (Figure 5). Although it is thought to play a large role in excluding *Medea* elements from India, the population dynamics of the H factor had not previously been investigated experimentally. While models of SGEs and their suppressors exist (e.g., Kobayashi & Telschow, 2010; Randerson, Smith, & Hurst, 2000), we do not know of any other studies examining their multigenerational dynamics in laboratory populations. Such studies are necessary for refining our understanding of the relationship between an SGE and its suppressor and of the individual elements themselves.

Here, we have found that M^1^ does not always behave as expected in the presence of H, as one population replicate saw a dramatic increase in the M^1^ element, despite the overwhelming presence of H individuals (Figure 5b). At the interface of *Medea* and H in India, matings between wild *Medea* and H‐bearing individuals are likely frequent, and a genetic factor lessening the severity of this interaction (or dismantling the functionality of *Medea* or H) would likely experience positive selection. However, additional studies in that region are needed to assess *Medea* and H frequencies, and whether a range of incompatibilities exist.

Differences in reproductive fitness between our strain bearing M^1^ and the H strain used in these population experiments were not directly assessed. We had previous difficulties in maintaining the H line, and this suggests a lower reproductive output. However, M^1^ declined in most replicates despite any possible reproductive advantage.

### 4.1 Insights for applications of *Medea* elements

We have presented here the first analyses of natural *Medea* dynamics in experimental populations. Further, this is the first study we are aware of to study a natural selfish element‐suppressor system in experimental populations. Suppressors of SGEs are of scientific interest not only because they impact the spread of the element, but because they can alter the evolutionary trajectory of a population. Because of the inherent conflict between SGEs and the host genome, suppressors of SGE components may be favored by natural selection (Hurst, 1995). The suppressors may either increase to fixation or be maintained with the SGE. If the SGE is neutralized, the suppressor may be coopted for other purposes. *Medea's* only currently known suppressor, H, is also interesting because it may represent a mechanism by which we could retroactively remove a drive mechanism from wild populations, should that be needed. We have demonstrated that a high frequency of H can sometimes purge M^1^ from a population. As H is currently thought to function by interfering with *Medea's* antidote system (Thomson, 2014), a synthetic H could function similarly, resulting in the death of offspring bearing the *Medea* construct, and impeding spread.

### 4.2 Comparisons with models

Low‐frequency M^1^ introductions typically fit model predictions without adding a heterozygote‐associated fitness costs, while higher‐frequency introductions were predicted slightly better by a model with these costs (Figures 3, 4, 5). This is largely because such fitness costs impede M^1^ increase at lower frequencies by eliminating the offspring of some heterozygotes but accelerate increase at higher frequencies through removing wild‐type allele‐carrying heterozygotes in an otherwise largely homozygous M^1^ population. These results suggest that, if a heterozygote fitness cost *does* exist, past models are missing another feature of M^1^ dynamics that could affect the increase in M^1^ frequency. Further quantification of this facet of *Medea* biology would be important for predicting *Medea's* behavior in pest‐management applications.

### 4.3 Future studies

Selfish genetic elements are found in a huge variety of taxa, and their spread can have important evolutionary consequence as well as innovative pest‐management applications. The study above is only a step toward understanding *Medea* dynamics. Our results make a good argument for the hypothesis that within a decade of our population sampling, *Medea* will have spread to more locations and increased in frequency. Two alternative hypotheses are that (a) a *Medea* suppressor will evolve over time as seen with another SGE in *Drosophila simulans* (Bastide et al., 2011) or (b) that once *Medea* becomes fixed in large geographic areas and is no longer driving, mutations will build up within the *Medea* sequences and the element will lose its capacity to drive. A follow‐up study of *Medea* frequencies would certainly be justified.

## CONFLICT OF INTEREST

The authors declare no financial conflict of interest.

## AUTHOR CONTRIBUTIONS

SAC, FG, and MDL designed the research; MDL provided reagents; SAC involved in performance of empirical research; SAC and MAR involved in performance of modeling; SAC and MAR analyzed the data; SAC, FG, and MDL interpreted the data; SAC involved in writing—original draft; and SAC, FG, and MDL involved in writing—review and editing.

## Data Availability

More data can be accessed in the following thesis: Cash (2016). An Experimental and Theoretical Analysis of the Selfish Genetic Element Medea in Red Flour Beetle Populations. PhD thesis, North Carolina State University, Raleigh. Data are archived on Dryad https://doi.org/10.5061/dryad.dfn2z34wr.
