# Supplementary material for: The impact of local population genetic background on the spread of the selfish element Medea‐1 in red flour beetles
Source: Ecol Evol. 2019 Dec 19;10(2):863–74. doi: 10.1002/ece3.5946 (PMC6988536; doi:10.1002/ece3.5946)

**Appendix S1 ---additional tables and figures**

**
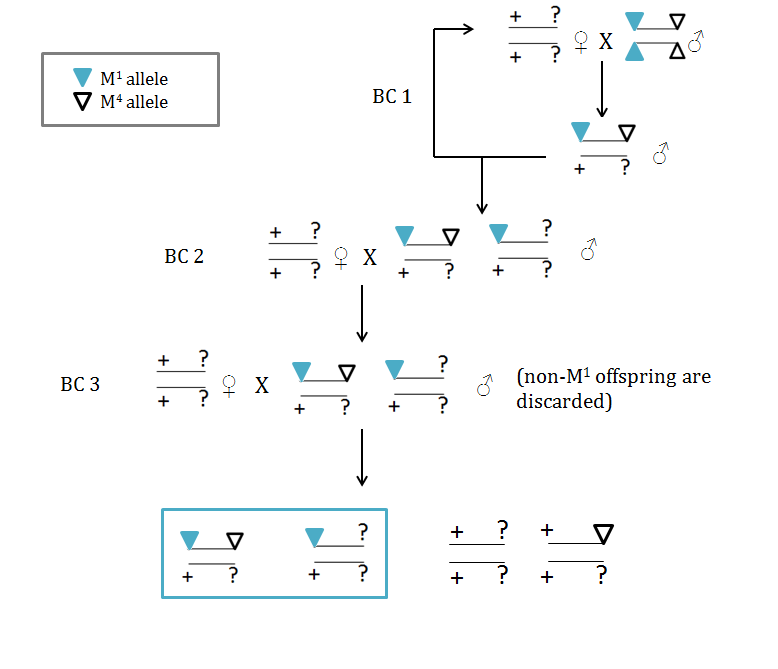
**

**Figure S1. Crossing M^1^ into a non-M^1^ population genetic background.** The M^1^ allele is indicated by a filled triangle, while M^4^ is represented by an open triangle. Question marks are present where M^4^ genotype is uncertain.

**
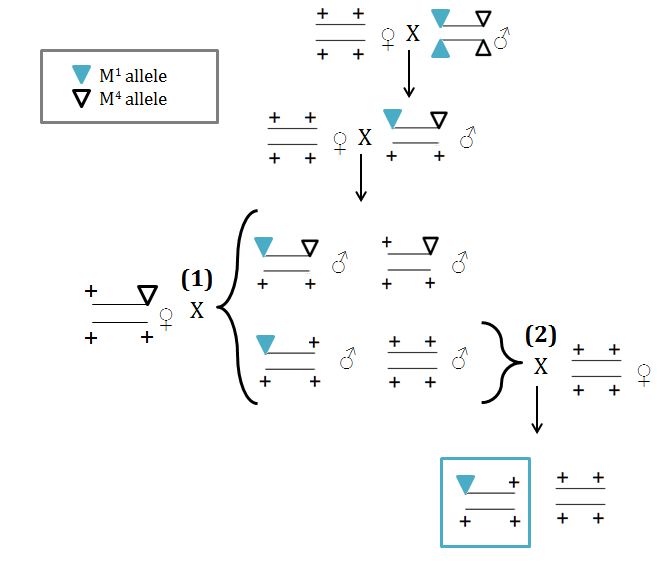
**

**Figure S2. Generation of an M^1^-only strain.** Crosses between M^1^- and M^4^-fixed Pig-19 and non-*Medea* GA-1 result in M^1^, M^4^ heterozygotes. Males are backcrossed to GA-1 non-*Medea* females. Male progeny are (1) crossed to heterozygous M^4^ females to diagnose M^4^ status. Non-M^4^ males are (2) retained and crossed to GA-1 females, then sacrificed and genotyped for M^1^. Progeny from crosses involving only M^1^-bearing, non-M^4^ males (grey box) are retained.

**(c)**

**(b)**

**(a)**

**Generation**

**Estimated M^1^ allele frequency**

**Figure S3. Comparison of intermediate-M^1^ population data and multiple models for (a) LA-4, (b) TX-3 and (c) TN-3 replicates.** Dotted lines represent models with a heterozygote fitness cost (0.3 for M^1^, 0.3 for M^4^), while dashed lines represent models with no cost. Models run with the low effective population size estimates (versus census population sizes) have larger standard deviations.

**(a)**

**(b)**

**Generation**

**Figure S4. Comparison of previously- fixed M^1^ population data, previously non-M^1^ population data, and multiple models.** Dashed lines represent models without fitness costs, while dotted lines represent models with a 0.3 *Medea* heterozygote fitness cost. In (b), black lines are AL-9-specific models, while grey lines are ND-1-specific (differ because of different expected M^4^ allele frequencies).

**Generation**

**(a)**

**(b)**

**Generation**

**Figure S5. Comparison of M^1^-H population data and both models.** Because fitness costs for heterozygous offspring of homozygous mothers are not expected to be a factor for populations with only M^1^-fixed and H-fixed individuals, only the model without fitness costs is shown in (a). In (b), the dashed line shows model expectations without fitness costs, while the dotted line shows expectations with a 0.3 M^1^ heterozygote cost.

**Appendix S2 Simulation Model Details**

All models used in this study were created in MATLAB, and all simulations were performed using MATLAB (Version 8.0.0.783, MathWorks, Natick, Massachusetts, USA).

***A single Medea element****:* We created a stochastic individual-based model of a single *Medea* element. Because both elements exhibit identical maternal-effect lethality, this model could represent a population harboring either M^1^ or M^4^. As with our experimental populations, generations are non-overlapping, and population size is constant.

First, the initial number of individuals of each genotype is selected from a multinomial distribution with sample size (N = 200) and probabilities equal to the initial genotype frequencies in the sample. In the case of a single *Medea*, three genotypes may exist in the population: MM (homozygous *Medea*), Mm (heterozygous *Medea*), and mm (non-*Medea*). When assessing the potential impact of a fitness cost imposed on the heterozygous offspring of homozygous *Medea* mothers, a fourth genotype is considered, where Mm offspring from MM mothers are treated separately to account for their different reproductive fitness (Xxxx 2016 Table S4.1a). Because there may be different fitness costs for males and females, we use *f* and *g* to impose a reproductive fitness costs on females and males, respectively. For example, *f* = 0.5 indicates that female Mm offspring of MM mothers produce roughly half as many eggs as their unaffected female counterparts. While we have not directly investigated the impact of either *Medea* element on male reproductive fitness, many SGEs are known to negatively impact male reproductive traits, including sperm quality, and so it is reasonable to consider the impact of such a male fitness cost on *Medea* increase (reviewed in Price and Wedell 2008.)

The number of females of each genotype is chosen from a binomial distribution, with probability equal to the initial female: male sex ratio (*r_f_*) in the sample. For example, at an *r_f_* of 0.4, the probability of a given individual being assigned as female is 0.4, and the probability is 0.6 that the individual will be assigned as male. Next, the number of times an individual female mates is chosen from a Poisson distribution with a mean of *λ_m_*. This is repeated for each female in the cohort. The genotype of a given female’s mate(s) is selected from a multinomial distribution with probabilities equal to the frequencies of the genotypes present in the male population. Pulling randomly (with replacement) from a frequency distribution of eggs per female derived from empirical egg-lay data gathered previously (Xxxx 2016 Figure S4.1), we generate the numbers of eggs that result from a mating. This random assignment of egg production repeats for each of a female's mates, so that the same female may have a range of mating successes, where a mating with one male may produce many offspring, but a mating with a subsequent male might produce none.

At this stage, the number of eggs may be decreased to represent a fitness cost based on parental genotype. For simulations of M^1^, the number of eggs produced by heterozygous offspring of homozygous M^1^ mothers is reduced based on empirical fitness data presented in (Xxxx 2016 Appendix C). For simulations of an allelic fitness cost, individuals with a single Medea allele contribute *m* offspring relative to wild-type individuals, where *m* represents a proportion between 0 and 1. Homozygous *Medea* individuals contribute *m*^2^. For example, with an *m* of 0.9, an Mm individual is roughly 90% as prolific as a wild-type individual, and an MM individual is roughly 81% as successful. This cost is applied to both males and females, such that an Mm x Mm pairing is roughly 81% as productive as a mating between two wild-type individuals.

Once the number of eggs per mating has been determined, the number of offspring of each genotype produced by each mating is chosen from a multinomial distribution, with probabilities determined by the probabilities of offspring genotypes produced from each mating. For instance, in a mating between two heterozygotes, the probability distribution of offspring assigned to MM, Mm and mm genotypes is (0.25, 0.5, 0.25), so that offspring have a 50% probability of assignment to the heterozygous genotype, and 25% probability of assignment to either MM or mm. Eggs assigned an inviable genotype (i.e., wild-type offspring of *Medea* mothers) are then eliminated at this stage, and do not contribute to the next generation. With probability equal to the desired sex ratio (typically 1:1), the number of offspring of each sex is chosen from a binomial distribution. We "sample" 200 random offspring to seed the next generation as before, where the number of individuals of each genotype is determined from a multinomial probability distribution with sample size N, and probabilities equal to the genotype frequencies of the offspring produced in the current generation. A generation-ending genotype distribution of 0.25 MM, 0.5 Mm, 0.25 mm would produce a probability distribution of (0.25, 0.5, 0.25) for selecting individuals with those genotypes. Thus, we would expect genotypes in our random sample to largely reflect true genotype frequencies in our simulated populations, but with some sampling variability.

If the model includes immigration, our sample size in the resident population changes to [N - (number of migrants)], such that new sources of either *Medea* or wild-type alleles are introduced to reproduce during the next generation. For example - in modeling a 5% immigration frequency and total population size of 200, 190 were randomly selected from the offspring of the prior generation, and 10 were migrants of known genotype. This is repeated each generation. At the beginning of the next generation, these migrants are subject to the same variation in reproductive frequency and quality as all other individuals.

***Two unlinked Medea elements***: We expanded the model described above, incorporating an additional, unlinked Medea locus. This reflects a real, biologically-relevant scenario, as the M^1^ element and M^4^ elements often co-occur in *Tribolium* strains (Beeman and Friesen 1999). One element cannot cross-rescue another - offspring of a *Medea* mother must inherit that same *Medea* element (or elements) to survive. Individuals may harbor either or both elements, and predicted offspring ratios assume free recombination between the two elements. Nine genotypes are possible under this scenario.

In order to assess the impact of fitness costs on the heterozygous offspring of homozygous *Medea* mothers, we expand the model to 16 genotypes - the nine original genotypes, plus seven heterozygous forms derived from crosses involving a homozygous *Medea* mother (Xxxx 2016 Table S4.1b). This allows us to impose a fitness cost only on these heterozygotes, and to allow the fitness cost to differ depending on whether the mother is homozygous M^1^, or homozygous M^4^, or homozygous for both elements. As before, we use *f* to designate the reproductive cost imposed on female offspring (i.e. "*f*_M1_ = 0.9" indicates that a heterozygous female offspring of a homozygous M^1^ mother produces an average of 90% as many offspring as an unaffected female). Similarly, we use *g* to designate the cost imposed on males (i.e. "*g*_M4_ = 0.75" indicates that matings involving a heterozygous male offspring of a homozygous M^4^ mother produce roughly 75% as many offspring as matings involving an unaffected male). In this model, when an affected heterozygote is the offspring of a doubly-homozygous mother, the fitness costs are multiplied - if *f*_M1_ is 0.9 and *f*_M4_ is 0.9, then *f*_M1M4_ is 0.81.

***Medea elements and the hybrid incompatibility factor (H)*:** We expanded on our basic *Medea* model to incorporate H-bearing genotypes. Though all nine possible genotypic combinations of M^1^ and H can occur in fertilized eggs, only five offspring genotypes are viable under this scenario: M^1^M^1^hh(homozygous M^1^, non-H), M^1^m^1^hh (heterozygous M^1^, non-H), m^1^m^1^HH (non-M^1^, homozygous H), m^1^m^1^Hh (non-M^1^ heterozygous H), m^1^m^1^hh (non-M^1^, non-H) (Xxxx 2016 Table S4.1c). As before, we add an additional genotype to our model (M^1^m^1^hh, derived from a homozygous M^1^ mother) to investigate fitness, with *f* and *g* as the female- and male-specific costs.

Interactions between M^4^ and H are more complex (Table S1). Because the severity of the M^4^-H interaction is greater at 25°C, largely mimicking the M^1^-H dynamics, we instead model M^4^-H interactions at 32°C, where *Medea*-H incompatibility is expected to be lessened, and viable hybrids are more likely (Thomson *et al.* 1995, Thomson and Beeman, 1999, Thomson 2014). Our model of M^4^-H dynamics includes twelve genotypes - the original nine M^4^-H genotypes, plus three heterozygous M^4^ genotypes considered separately to investigate fitness costs of M^4^ heterozygotes (Xxxx 2016 Table S4.1d). As before, *f* and *g* indicate the female-specific and male-specific costs.

We include two additional variables in the M^4^-H model. At 32°C, a proportion of offspring (roughly 25-50%, dependent on parental genotypes) survive from crosses involving an M^4^ (non-H) mother and an H father, possibly due to a decrease in inhibitory interactions between H and the M^4^ antidote at this higher temperature (Thomson et al. 1995, Thomson 2014). Here, we use *j* to represent the percent survival of H-bearing offspring from these crosses, ranging from *j* = 0, where all offspring die, to *j* = 1, where all survive. From Thomson (2014), it was demonstrated that M^4^ loses maternal-effect lethality when the mother carries both M^4^ and H, so that wild-type offspring of M^4^m^4^, H-bearing mothers are rescued. Here, *k* represents the percent rescue of wild-type offspring of these mothers, where *k* = 0 represents normal maternal-effect lethality (complete lethality; all wild-type offspring of these mothers die) to *k* = 1, where all wild-type offspring are rescued.

**Table S1. Viable offspring genotypes according to maternal genotype.** Viability of a particular progeny genotype is indicated by the presence of a beetle in the corresponding cell. Empty cells represent inviable genotypes, due to either *Medea*-dependent lethality or the hybrid incompatibility factor. Genotypes represent both the homozygous or heterozygous Medea and H states (i.e., "M^1^" applies to individuals homozygous OR heterozygous for the M^1^ element). Table created by author with data from Thomson and Beeman 1999, Thomson 2014.


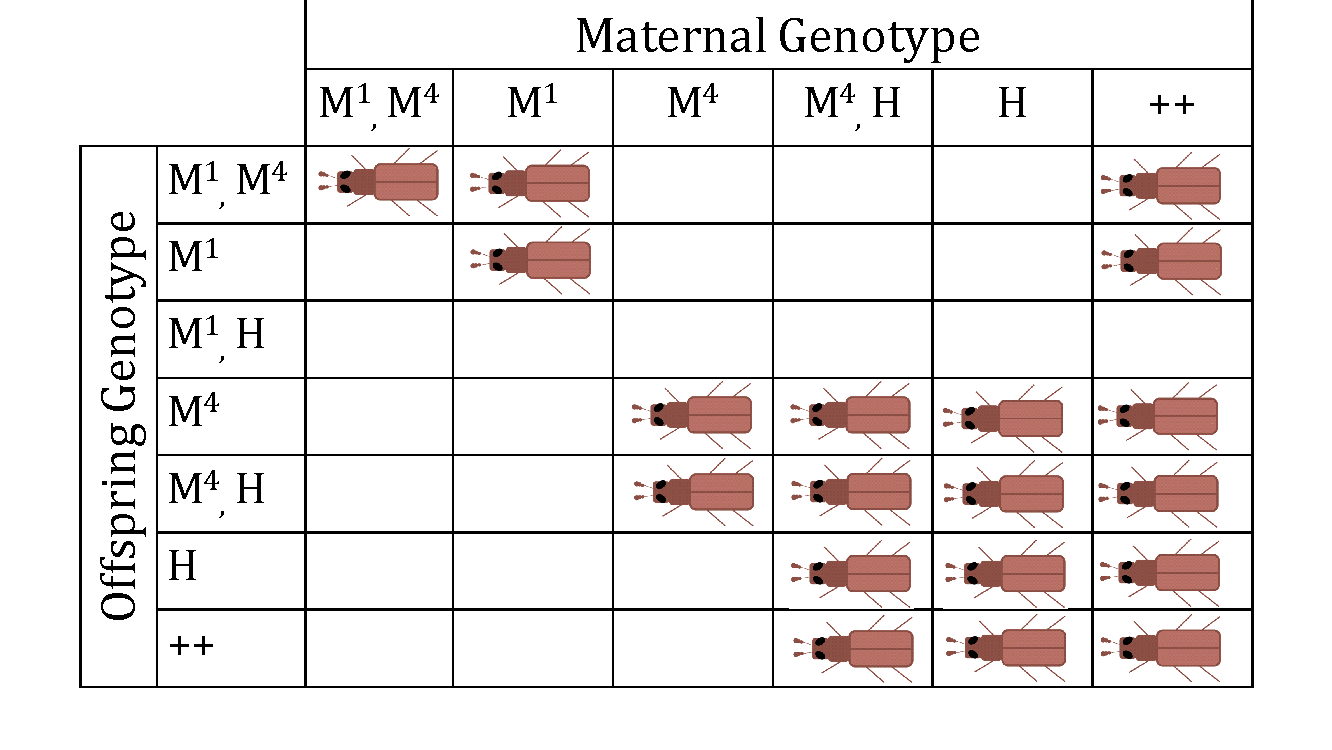

Supplement: Supplementary file 1 [file ECE3-10-863-s001.docx]
